# Phylogenetic Analysis of Human Rhinovirus Isolates Collected from Otherwise Healthy Children with Community-Acquired Pneumonia during Five Successive Years

**DOI:** 10.1371/journal.pone.0080614

**Published:** 2013-11-19

**Authors:** Cristina Daleno, Antonio Piralla, Alessia Scala, Laura Senatore, Nicola Principi, Susanna Esposito

**Affiliations:** 1 Pediatric Highly Intensive Care Unit, Department of Pathophysiology and Transplantation, Università degli Studi di Milano, Fondazione IRCCS Ca’ Granda Ospedale Maggiore Policlinico, Milan, Italy; 2 Virology Unit, Fondazione IRCCS Policlinico San Matteo, Pavia, Italy; The University of Hong Kong, China

## Abstract

In order to evaluate the circulation of the different human rhinovirus (HRV) species and genotypes in Italian children with radiographically confirmed community-acquired pneumonia (CAP), a nasopharyngeal swab was obtained from 643 children admitted to hospital because of CAP during five consecutive winter and early spring seasons (2007-2012). Real-time reverse transcriptase polymerase chain reaction (RT-PCR) was used to identify HRV, and the HRV-positive samples were used for sequencing analysis and to reconstruct the phylogenetic tree. HRV was identified in 198 samples (42.2%), and the VP4/VP2 region was successfully amplified in 151 (76.3%). HRV-A was identified in 78 samples (51.6%), HRV-B in 14 (9.3%) and HRV-C in 59 (39.1%). Forty-seven (31.1%) of the children with HRV infection were aged <1 year, 71 (47.0%) were aged 1-3 years, and 33 (21.9%) were aged ≥4 years. Blast and phylogenetic analyses showed that the HRV strains were closely related to a total of 66 reference genotypes, corresponding to 29 HRV-A, 9 HRV-B and 28 HRV-C strains. Nucleotide variability was 37% between HRV-A and HRV-B, 37.3% between HRV-A and HRV-C, and 39.9% between HRV-B and HRV-C. A number of sequences clustered with known serotypes and, within these clusters, there were strains circulating during several seasons. The most frequently detected genotypes were HRV-A78 (n=17), HRV-A12 (n=9) and HRV-C2 (n=5). This study shows that, although it is mainly associated with HRV-A, pediatric CAP can also be diagnosed in subjects infected by HRV-C and, more rarely, by HRV-B. Moreover, a large number of genotypes may be involved in causing pediatric CAP and can be different from year to year. Although the prolonged circulation of the same genotypes can sometimes be associated with a number of CAP episodes in different years.

## Introduction

The use of molecular methods of respiratory viral screening has recently made it possible to establish that human rhinoviruses (HRVs) are not only the main cause of the common cold (as thought since they were first identified several decades ago), but also common etiologic agents of lower respiratory tract infections (LRTIs) such as bronchiolitis and community-acquired pneumonia (CAP) [1-6]. It has also been reported that they are the pathogens most frequently associated with asthma exacerbations [7,8]. These findings have significantly increased interest in better defining HRV epidemiology mainly in order to clarify the possible association between specific strains and the development of more severe respiratory problems. 

A number of studies have reported epidemiological data showing HRV infection rates in children and adults [4-6,9-12]. It has been generally found that various HRV genotypes simultaneously circulated in a given period of time in a specific geographic area, most of which belonged to the A and C species; furthermore, the genotypes belonging to species A were mainly associated with CAP and those belonging to species C were mainly associated with wheezing. However, most of these studies were carried out in a single year and involved a relatively small number of patients, and only a few analysed specific LRTIs. Consequently, there are few data concerning the circulation of HRVs over a long period of time or the real role of the different species and genotypes in causing LRTIs. 

The aim of this study was to evaluate the circulation of the different HRV species and genotypes in Italian children with radiographically confirmed CAP during the winter and early spring of five consecutive years as this information could help to develop tailored strategies for the prevention and treatment of pediatric HRV infections.

## Material and Methods

### Study population and samples

The study was carried out in Pediatric Clinic 1 of the Department of Pathophysiology and Transplantation of the University of Milan during five consecutive years. The enrolment occurred between November 1 and April 30 in the years 2007-2008, 2008-2009, 2009-2010 and 2011-2011 and between November 1 and June 30 in 2011-2012. It was approved by the Institutional Review Board of the Fondazione IRCCS Ca’ Granda, Ospedale Maggiore Policlinico, Milan, Italy. The written informed consent of a parent or legal guardian was required, and the older children were asked to give their assent. 

All of the children aged between one month and 14 years seen in the Emergency Room (ER) of the Department of Pathophysiology and Transplantation who had fever (defined as an axillary temperature of >38°C), signs and symptoms of lower respiratory tract infection (i.e. cough, tachypnea, dyspnea or respiratory distress, and breathing with grunting or wheezing sounds with rales) and a chest radiograph consistent with CAP (in accordance with the World Health Organization criteria for the standardized interpretation of pediatric chest radiographs for a diagnosis of pneumonia [13]) were considered eligible for the study. The exclusion criteria were chronic diseases increasing the risk of respiratory infections, including premature birth; chronic disorders of the pulmonary or cardiovascular systems, including asthma; chronic metabolic diseases, including diabetes mellitus; neoplasia; kidney or liver dysfunction; hemoglobinopathies; immunosuppression; diseases requiring long-term aspirin therapy; and genetic or neurological disorders. The children with presumed nosocomial CAP (i.e. appearing more than 48 hours after admission or within two weeks of hospital discharge) were also excluded. A sample of nasopharyngeal secretions was collected from all of the children with radiographically confirmed CAP using a flexible pernasal flocked swab that was immediately placed in a mini-tube containing 1 mL of universal trasport medium (UTM-RT Kit Cat. No. 360c, Copan Italia, Brescia, Italy). 

### Nucleic acid extraction and real-time reverse transcriptase polymerase chain reaction (RT-PCR)

Viral nucleic acids were extracted from the nasopharyngeal swabs using a Nuclisens EasyMAG automated extraction system (Biomeriéux, Craponne, France), and the extracts were tested for respiratory viruses using the RVP Fast assay (Luminex Molecular Diagnostics Inc., Toronto, Canada) in accordance with the manufacturer’s instructions. The RVP Fast assay consists of a single multiplex polymerase chain reaction (PCR) with labelled primers, followed by the single-step hybridization of the PCR products with the fluorescent bead array and incubation with reporter reagents. The plate was analysed using a Bio-Plex 200 System (Bio-rad Laboratories, Milan, Italy) and its associated software Luminex x PONENT version 3.1 (Luminex Molecular Diagnostics Inc., provided by Abbott), and the median fluorescent intensity (MFI) was determined. An MFI above the threshold level determined by the manufacturer for a particular target indicated a positive result for that target. The mean positive fluorescence intensities were established using Tag-It Data Analysis Software (TDAS, Luminex). The RVP Fast assay simultaneously detects influenza A virus (subtyped H1 or H3), influenza B virus, respiratory syncytial virus (RSV)-A and -B, parainflunzavirus-1, -2, -3 and −4, adenovirus, human metapneumovirus (hMPV), coronaviruses 229E, NL63, OC43 and HKU1, enterovirus/rhinovirus, and human bocavirus. The samples that were positive for enterovirus/rhinovirus were retested by means of one-step real-time RT-PCR in order to identify the rhinovirus [6]. 

### Sequencing analysis, phylogeny and classification

The hypervariable part of the 5' NCR (non-coding region), the entire VP4 gene and the 5' terminus of the VP2 gene in the HRV-positive samples were amplified by means of a RT-PCR as previously described [6,14]. The PCR products were purified using the Wizard SV Gel and PCR Clean-Up System (Promega, Milan, Italy), and then sequenced in both directions using the same forward and reverse primers as those used in the PCR. The nucleotide sequences were obtained by means of automated DNA sequencing using an ABI PRISM 3730 genetic analyser (Applied Biosystems, Foster City, CA). The newly determined sequences were edited using Sequencher software, and aligned using the ClustalW program integrated in the MEGA version 5.0 package [15]. The analysed fragment was 393 nt in the VP4/VP2 region. The HRV in each sample was determined on the basis of the phylogenetic tree and by comparing it with all of the available rhinovirus reference prototypes encoding VP4/VP2 protein retrieved from GenBank (http://www.ncbi.nlm.nih.gov). The phylogenetic tree was reconstructed using the neighbour-joining method and parameters selected by the MEGA model test program. Branch support was assessed by means of bootstrap analysis with 1000 replicates. Genotypes were assigned on the basis of their clustering with known prototype reference strains present in GenBank. The genetic distances between and within HRV-A, HRV-B, HRV-C were tested using the MEGA program and compared by means of Student’s *t*-test. The graphs were made using GraphPad Prism version 5.01 for Windows (GraphPAD Software, San Diego, CA).

### Data collection and nucleotide sequence data set

The 151 VP4/VP2 sequences described in this study have been deposited in Genbank under accession numbers KF427709 - KF427859.

## Results

### HRV types

During the five years of the study, 643 children with radiographically confirmed CAP were enrolled. Of these, 469 (72.9%) were positive for at least one virus, and 174 (27.1%) were negative. HRV was identified in 198 samples (42.2%), in 60% of the cases as single infectious agent. The VP4/VP2 region was successfully amplified in 151 (76.3%); the remaining 47 samples (23.7%) were excluded from the phylogenetic analysis because of poor sequence quality. All three HRV types were circulating in the study population: type HRV-A was identified in 78 samples (51.6%), HRV-B in 14 (9.3%), and HRV-C in 59 (39.1%). 

As shown in Table 1, 47 (31.1%) of the children with HRV infection were aged <1 year, 71 (47.0%) were aged 1-3 years, and 33 (21.9%) were aged ≥4 years. HRV-A and HRV-C were equally frequent in the patients aged <1 year (44.7% *vs* 42.6%), whereas their incidence was different in the children aged ≥4 years (57.6% *vs* 30.3%). HRV-B was the less frequently species in all three age categories. 

**Table 1:** Incidence of HRV infections in different age groups of children with community-acquired pneumonia (CAP).

	**No. of samples**	**No. (%) of positive samples**		
**Age group**	**n=151**	**HRV-A**	**HRV-B**	**HRV-C**
< 1	47	21 (44.7%)	6 (12.8%)	20 (42.6%)
1-3	71	39 (54.9%)	4 (5.6%)	28 (39.4%)
> 4	33	19 (57.6%)	4 (12.1%)	10 (30.3%)

**Figure 1:**
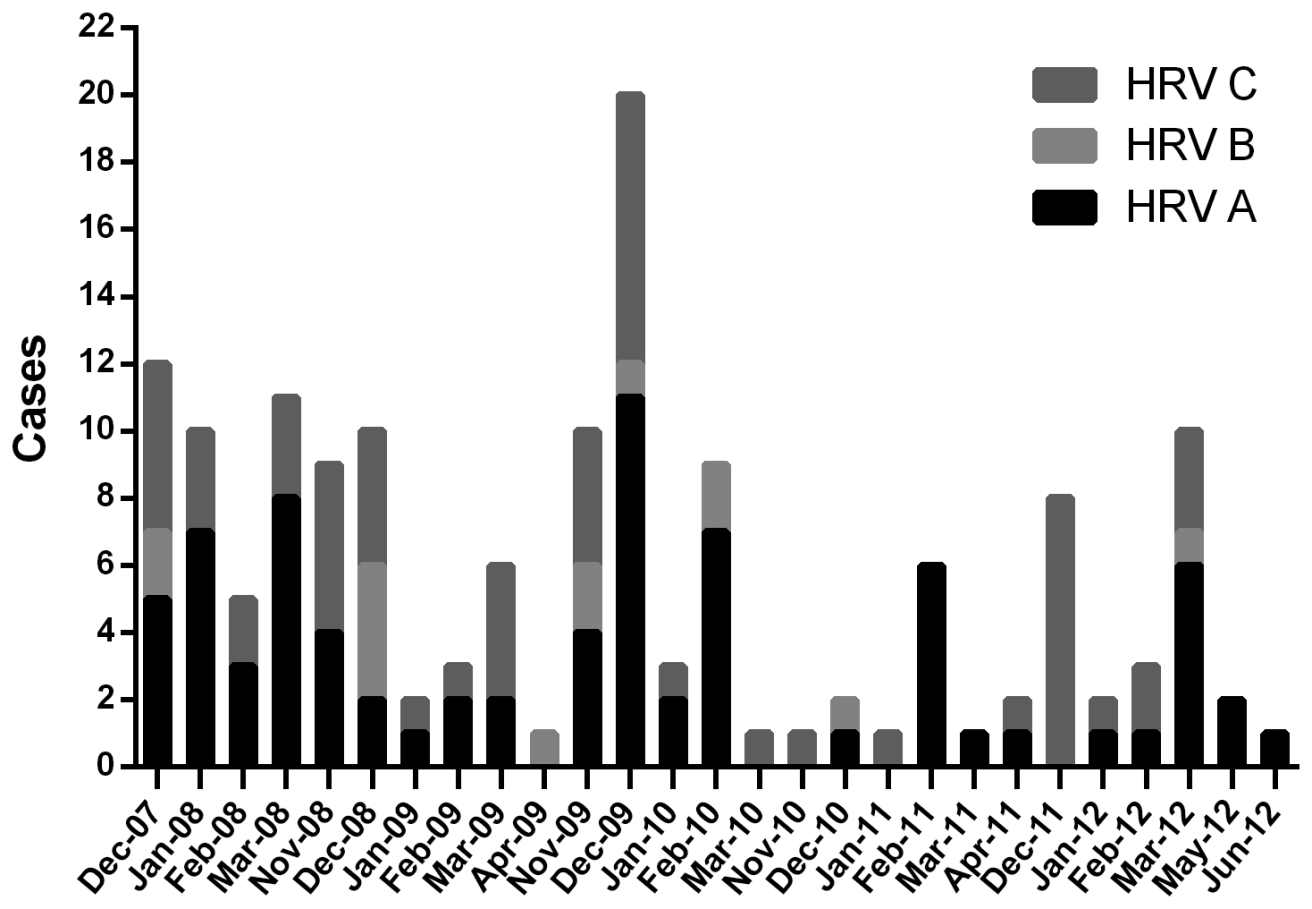
Distribution of HRV species over a 5-year period in children with community-acquired pneumonia (CAP) admitted to the Department of Pathophysiology and Transplantation, University of Milan, Milan, Italy. The Figure reported the months in which the HRV species were identified during the study periods.

Figure 1 shows the monthly distribution of the HRV species over the 5-year study period. HRV-A and HRV-C was circulating in almost all of the months of surveillance without any clear seasonal pattern: HRV-A cases were frequently observed in February (20/26; 76.9%) and March (17/29; 58.6%), whereas HRV-C cases were frequently observed in December (25/52; 48.1%). HRV-B occurred only sporadically throughout the study period.

### Genetic variations and clustering of strains

**Figure 2:**
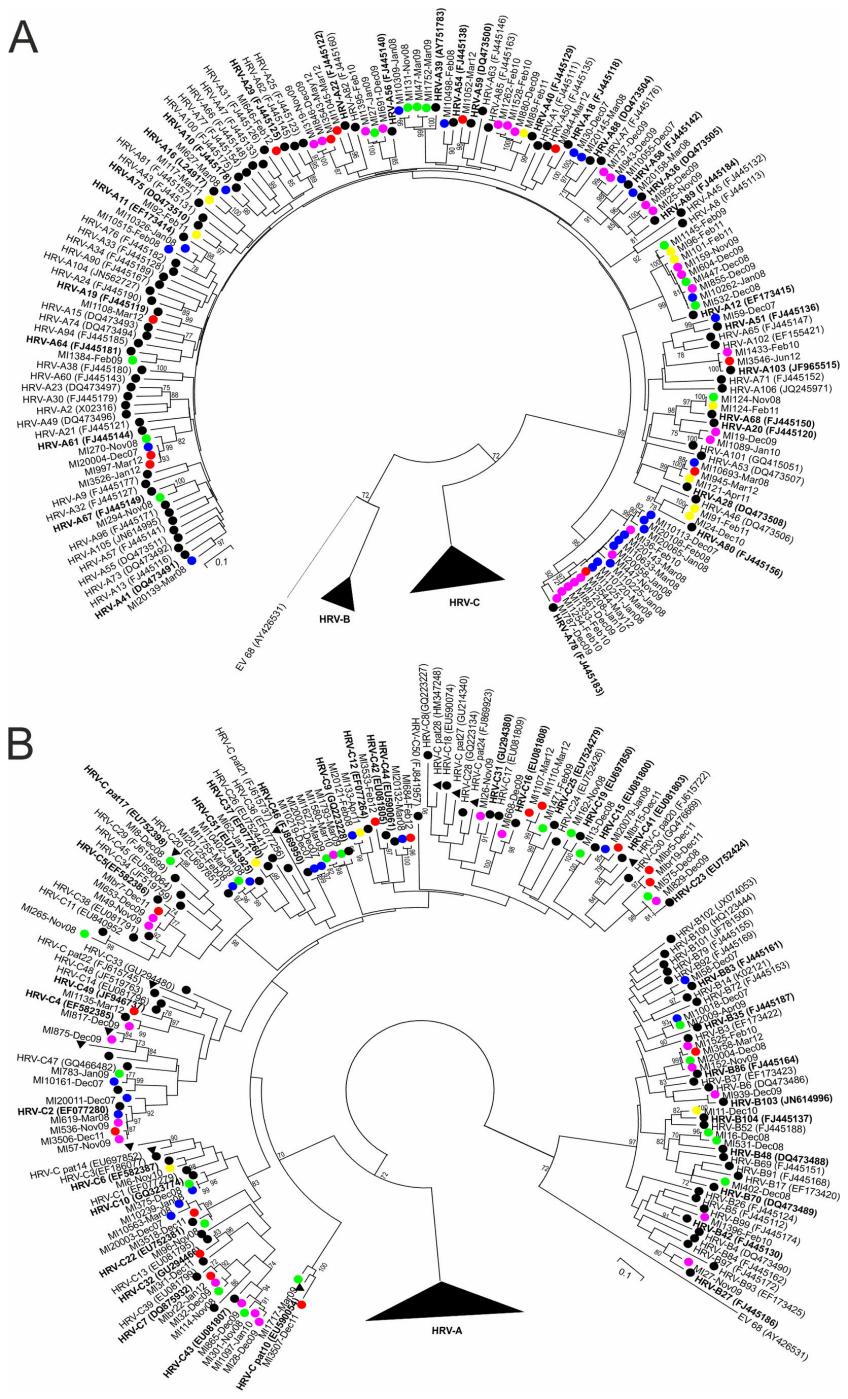
Phylogenetic tree based on neighbour-joining analysis of 78 HRV-A VP4/VP2 (A) and 14 HRV-B and 59 HRV-C VP4/VP2 (B) sequences from the present study and database reference strains. The coloured dots indicate the sampling season: blue, 2007-2008; green, 2008-2009; pink, 2009-2010; yellow, 2010-2011; red, 2011-2012; black, reference strains. Black triangles, provisionally assigned types (pats) of HRV-C.

On the basis of blast and phylogenetic analyses, the Italian HRV strains were closely related to a total of 66 reference genotypes corresponding to 29 HRV-A, 9 HRV-B and 28 HRV-C strains. Table 2 shows the frequencies of the HRV genotypes detected in each season during the study period. The HRV sequences showed marked genetic diversity. The HRV-C sequences were the most heterogenous, with an intra-species nucleotide *p*-distance of 0.25, which was greater than the *p*-distance within HRV-A (0.20) or HRV-B (0.21). Nucleotide variability was 37% between HRV-A and HRV-B, 37.3% between HRV-A and HRV-C, and 39.9% between HRV-B and HRV-C. Figure 2 shows the phylogenetic tree constructed on the basis of the VP4/VP2 region of the 151 sequences from this study and the references sequences. A number of sequences clustered with known genotypes and, within these clusters, there were strains circulating during several seasons. The most frequently detected types were HRV-A78 (n=17), HRV-A12 (n=9) and HRV-C2 (n=5). 

**Table 2:** HRV genotypes within each species detected among study subjects.

	**No. of sample with the indicated species and genotype**					
**Species and genotype**	**Total**	**07-08**	**08-09**	**09-10**	**10-11**	**11-12**
**A78**	17	9		7		1
**A12**	9	1	3	3	2	
**A22**	4			2		2
**A39**	4	1	3			
**A40**	4			3	1	
**A61**	4	1	1			2
**A58**	3	1		2		
**A28**	3	1			1	1
**A56**	3		1	2		
**A89**	2			2		
**A80**	2				2	
**A20**	2			2		
**A88**	2	2				
**A68**	2		1		1	
**A11**	2	2				
**A103**	2			1		1
**A54**	1	1				
**A59**	1					1
**A29**	1					1
**A75**	1				1	
**A67**	1		1			
**A18**	1					1
**A41**	1	1				
**A51**	1	1				
**A36**	1	1				
**A10**	1	1				
**A16**	1				1	
**A64**	1		1			
**A19**	1					1
**Total HRV-A**	**78**	**23**	**11**	**24**	**9**	**11**
**B86**	4		1	2		1
**B48**	2		2			
**B35**	2	1	1			
**B42**	1			1		
**B27**	1			1		
**B70**	1		1			
**B83**	1	1				
**B103**	1			1		
**B104**	1				1	
**Total HRV-B**	**14**	**2**	**5**	**5**	**1**	**1**
**C2**	5	2		2		1
**C23**	4		1	1		2
**C43**	4		1	3		
**C22**	4	2	1			1
**C25**	3		1			2
**C7**	3		1	1		1
**C9**	3		2	1		
**C5**	3			2		1
**C51**	3	2	1			
**C41**	2	1				1
**C12**	2	1			1	
**C40**	2	1		1		
**C10**	2	1	1			
**C46**	2	2				
**C44**	2	1				1
**C? (pat10)**	2		1			1
**C37**	1				1	
**C15**	1		1			
**pat17**	1		1			
**C42**	1					1
**pat19**	1			1		
**C32**	1					1
**C6**	1				1	
**C31**	1			1		
**C16**	1			1		
**C27**	1		1			
**C4**	1			1		
**C49**	1					1
**C19**	1		1			
**Total HRV-C**	**59**	**13**	**14**	**15**	**3**	**14**

### HRV-A

**Figure 3:**
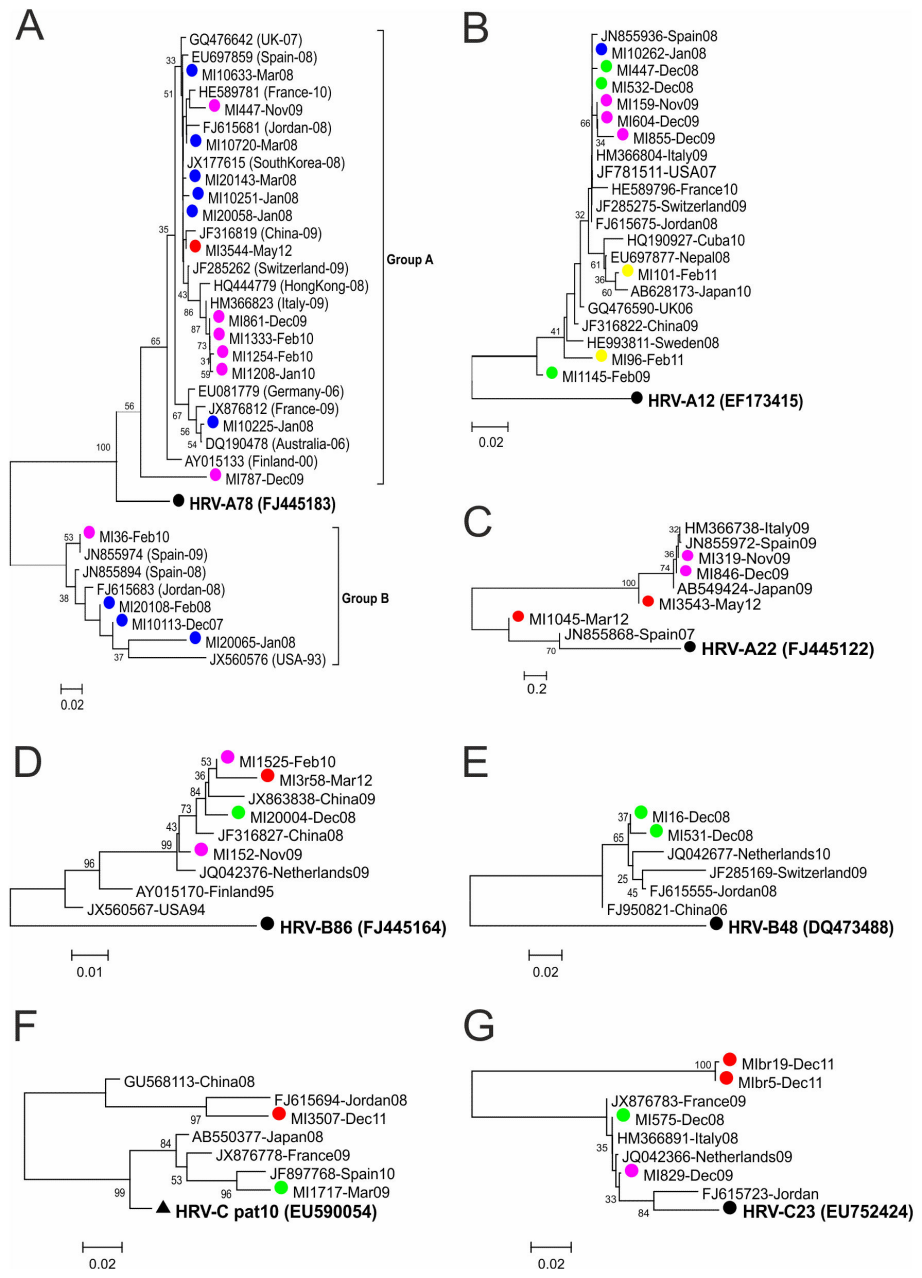
Phylogenetic tree based on neighbour-joining analysis including Italian HRV-A78 (A), HRV-A12 (B), HRV-A22 (C), HRV-B86 (D), HRV-B48 (E), HRV-Cpat10 (F), HRV-C23 (G)-like strain sequences in comparison with the most closely related sequences found in GenBank. The coloured dots indicate the sampling season: blue, 2007-2008; green, 2008-2009; pink, 2009-2010; yellow, 2010-2011; red, 2011-2012; black, reference strains. Black triangles, provisional assigned types (pats) of HRV-C.

HRV-A78-like strains were observed during three alternate seasons (2007-08, 2009-10, 2011-12). The Italian HRV-A78 sequences seem to cluster into two different groups (A and B, Figure 3A): in comparison with the reference strain, mean nucleotide divergence in cluster B was significantly greater than in cluster A (10.2% *vs* 6.9%; p<0.001). 

The HRV-A12 strain circulated in four consecutive seasons (2007-2011). Six of the nine HRV-A12-like sequences (66.6%) showed high degree of identity (mean 99%) in comparison with that between them and the reference strain (mean 92%); the remaining three strains were dispersed with HRV-A12-like strains circulated worldwide (Fig. 3B). It is worth noting that two identical HRV-A12 strains were observed in two different seasons (MI10262-Jan08 and MI532-Dec08), and the same strains were also found in the USA in 2007, and Italy and Switzerland in 2009. 

HRV-A22-like strains clustered in two different groups with respectively 8.2% and 11.6% nucleotide divergence from the reference strain. These sequences circulated in two non-consecutive seasons (2009-10 and 2011-12); however, one strain found in 2011-2012 (MI3543) showed a greater degree of nucleotide similarity with two HRV-A22-like sequences circulating in 2009-2010 than with another strain circulating in the same year. This could mean that the HRV-A22-like strains remained in the studied population for a long time, probably more than two years (Figure 3C).

An identical HRV-A68-like strain was identified in samples taken during two non-consecutive seasons (MI124-Nov08 and MI124-Feb11). In addition, a series of HRV-A-like sequences showed a nucleotide divergence of >10.5% from their reference strains: MI19 and MI1089 *vs* HRV-A20, and MI24 and MI91 *vs* HRVA-80 (Figure 2A).

### HRV-B

All of the sequences defined as being HRV-B86-like and HRV-B48-like strains showed a nucleotide divergence of >9.5% from the reference strains (mean 9.9% and 11.6%) (Figures 3D and 3E). HRV-B86-like strains were the most frequent B genotype, and circulated in three different seasons. HRV-B35-like strains were found in two different seasons with the presence of the same strain in both years (MI10018-Dec07 and MI2009-Apr09), and a nucleotide divergence of 8.4% from the reference strain. 

### HRV-C

Two strains (MI1717-Mar09 and MI3507-Dec11) clustered alone without any reference strains (Figure 2B). The nucleotide identity of the two sequences with HRVC pat10 (EU590054, which is not currently assigned to genotypes) was >90% (Figure 3 F). 

The four HRVC-23-like strains were all observed in December of three different seasons. Two of them (MIbr19-Dec11 and MIbr5-Dec11) showed significantly greater nucleotide divergence from the reference strain than the other two strains (MI829-Dec09 and MI575-Dec08) (mean 11.7% and 4.2%; p<0.001), which suggests that they may belong to a new HRV-C genotype (Figure 3G). However, the sequencing of VP1 for these two strains is required for type assignment.

The MI8-Dec08 and MI875-Dec09 sequences showed more than 10.5% nucleotide divergence (14.2% and 12.9%, respectively) from their most similar reference strains (HRV-C29 and HRV-C35). These two sequences respectively grouped with the unassigned serotype sequences HRVC pat17 (EU752398) and HRVC pat19 (FJ598096) (Figure 2B).

## Discussion

This study is based on five years’ surveillance of HRV infection in Italian children with radiographically confirmed CAP and confirms the high frequency of the association between this virus and pediatric CAP. The presence of a virus in the nasopharynx of a child with CAP does not necessarily mean that it is the etiological agent of the disease because it may only indicate a coincidental upper airways infection, or be due to a carrier state or the prolonged shedding of a pathogen that caused a previous infection. This may be particularly important in the case of RVs because a number of epidemiological studies have shown that they can be found in the respiratory secretions of 12–22% of asymptomatic subjects [16,17]. However, in our study population, we found HRV as a single agent in about 60% of the children in which this pathogen was identified. This strongly suggests that it was the cause of the CAP diagnosed in many of our study patients, particularly in those in whom very low values of WBC counts and CRP were found. 

The most frequently detected HRV species was HRV-A (51.6%), followed by HRV-C (39.1%) and HRV-B (9.3%). These detection rates are similar to those recently reported in various countries [18-20], but different from those of Calvo et al. [21] and Linsuwanon et al. [22], who studied patients with LRTIs and found that HRV-B was predominant when CAP was diagnosed. However, the diagnosis of CAP in the last two studies was not always radiographically confirmed and this could have affected the results. 

We detected HRVs more frequently in children aged <1 year (31.1%) and in those aged 1-3 years (47%), than in those aged ≥4 years (21.9%), as has been reported in other studies [18,23]. In terms of species, HRV-A was more frequently detected than HRV-C in children aged 1-3 and ≥4 years, although a recent study in Thailand found that HRV-C was the most frequent species in children with LRTIs in the same age groups [24]. In our study the circulation of HRV was evaluated only during the winter and spring months. This means that data do not indicate the real circulation of HRV species during the whole year. However, ours is the first study defining the circulation of HRV species and genotypes in children with CAP that is based on such a large number of samples collected over a 5-year observation period, which is significantly longer than in other studies [19,25-28] and can give a precise information on the circulation in time of the different HRV species. . 

The HRV-positive samples contained a total of 66 genotypes (29 HRV-A, nine HRV-B and 28 HRV-C), which is quite similar to the number reported in a recent 4-year study of children and adults with LRTIs in Sweden [29], but higher than the 45 genotypes found in a study carried out in Central Italy [27]. However, the observation period of this study was limited to one year and only a small number of CAP patients were enrolled. 

We classified the HRV genotypes using VP4/VP2-based phylogenetics, and distinguished the inter- and intra-serotypes using the nucleotide divergence threshold very recently proposed by McIntyre et al.: 10.5% for HRV-A, 9.5% for HRV-B, and 10.5% for HRV-C [30]. However, in the absence of a VP1 sequence, the putative new types are considered provisionally assigned. Our data show that a large number of genotypes can circulate during each observation period, and that some can circulate in more than one year and simultaneously in different geographic areas. This is clearly shown by the similarity between the Italian HRV-A strains identified in the present study and those found in Spain [31] and Jordan [32] during 2008, and the fact that we also found the genotypes most frequently detected in Sweden by Sansone et al.: A78, A12, A56, A10, C9 and C43 [29]. Considering only the 2009-2010 season, genotypes A12, A40, A89 and C2 found in this study were also found in a study carried out in France and involving a population similar to ours [33]. Furthermore, HRV-A12 was the most common genotype found in children with a primary diagnosis of viral CAP in Beijing, China, during the 2007-2008 season [34].

There was a wide range of genotypes with different degrees of nucleotide variations from their reference strain. Increased nucleotide variability with respect to prototype strains has been observed in studies carried out all over the world [20,26,29,34-36]. The considerable attention given to sequencing over the last few years and the development of efficient molecular methods capable of characterising HRV species and genotypes will allow the discovery of new variants in the near future.

## Conclusions

Our findings show that, although CAP is mainly associated with HRV-A, it can also be diagnosed in subjects infected by HRV-C and, more rarely, HRV-B. Our analysis of the genotypes associated with pediatric CAP suggests that a large number of genotypes may be involved in causing the disease. These may vary from year to year although the prolonged circulation of the same genotypes can sometimes be associated with a number of CAP episodes in different years. This could be the case of HRV-A78-like and HRV-A12-like strains, which were the most frequently found in this study. However, the importance of these types and the need to develop preventive measures against them should be confirmed in further studies of larger populations from different parts of the world. Further studies are also needed to evaluate whether any of the different types of HRV is more frequently associated with bacterial superinfection. Finally, it could be useful to analyse their ability to cause viremia, which has been recently associated with more severe HRV disease [37]. 
